# Eosinophils, but Not Type 2 Innate Lymphoid Cells, Are the Predominant Source of Interleukin 4 during the Innate Phase of *Leishmania major* Infection

**DOI:** 10.3390/pathogens11080828

**Published:** 2022-07-25

**Authors:** Carolin Sasse, David Barinberg, Stephanie Obermeyer, Andrea Debus, Ulrike Schleicher, Christian Bogdan

**Affiliations:** 1 Mikrobiologisches Institut—Klinische Mikrobiologie, Immunologie und Hygiene, Universitätsklinikum Erlangen und Friedrich-Alexander-Universität (FAU) Erlangen-Nürnberg, Wasserturmstraße 3/5, D-91054 Erlangen, Germany; carolin.sasse@uk-erlangen.de (C.S.); david.barinberg@uk-erlangen.de (D.B.); stephanie.obermeyer@gmx.de (S.O.); andrea.debus@uk-erlangen.de (A.D.); 2 Medical Immunology Campus Erlangen, FAU Erlangen-Nürnberg, Schlossplatz 4, D-91054 Erlangen, Germany

**Keywords:** eosinophils, interleukin-4, innate lymphoid cells (ILC), ILC2, leishmaniasis, *Leishmania major*, type 2 immune response

## Abstract

Interleukin (IL)-4 plays a central role in the initiation of a type 2 T helper cell (Th2) response, which leads to non-healing and progressive infections with the protozoan parasite *Leishmania* (*L.*) *major*. Here, we tested the hypothesis that type 2 innate lymphoid cells (ILC2), which promote the development of Th2 cells, form an important source of IL-4 early after intradermal or subcutaneous *L. major* infection. Lineage-marker negative CD90.2^+^CD127^+^PD1^−^ ILC2 were readily detectable in the ear or foot skin, but hardly in the draining lymph nodes of both naïve and *L. major*-infected self-healing C57BL/6 and non-healing BALB/c mice and made up approximately 20% to 30% of all CD45^+^SiglecF^−^ cells. Dermal ILC2 of C57BL/6 mice expressed the inducible T cell-costimulator (ICOS, CD278), whereas BALB/C ILC2 were positive for the stem cell antigen (Sca)-1. Within the first 5 days of infection, the absolute numbers of ILC2 did not significantly change in the dermis, which is in line with the unaltered expression of cytokines activating (IL-18, IL-25, IL-33, TSLP) or inhibiting ILC2 (IL-27, IFN-γ). At day 5 to 6 post infection, we observed an upregulation of IL-4, but not of IL-5, IL-10 or IL-13 mRNA. Using IL-4-reporter (4get) mice, we found that the production of IL-4 by C57BL/6 or BALB/c mice was largely restricted to CD45^+^SiglecF^+^ cells of high granularity, i.e., eosinophils. From these data, we conclude that eosinophils, but not ILC2, are a major innate source of IL-4 at the skin site of *L. major* infection.

## 1. Introduction

*Leishmania* are protozoan pathogens that are transmitted by sand fly vectors and cause a spectrum of local cutaneous, mucocutaneous or visceral diseases in mammalian organisms, including humans and mice [1,2]. The course of infection is determined by the parasite species and by the innate and adaptive immune response of the infected host organisms [3,4,5]. Over the past decades, experiments with the C57BL/6 mouse strain have yielded valuable insights into the key cellular, soluble and enzymatic factors that are critical for the control of *Leishmania (L.) major*, a parasite species that elicits cutaneous leishmaniasis [6]. The resolution of disease depends on dendritic cells [7,8] and type 1 T helper cells (Th1) [3,5], which release interferon (IFN)-γ and tumor necrosis factor (TNF) and thereby cause classical macrophage activation characterized by the expression of inducible or type 2 nitric oxide (NO) synthase (iNOS, NOS2) [9]. Additional protective effects are conveyed by the activation of natural killer (NK) cells [10,11] and by the generation of reactive oxygen species via the phagocyte NADPH oxidase [9].

Unlike C57BL/6 mice, BALB/c mice develop non-healing and progressive cutaneous as well as visceral disease following *L. major* infection. Consequently, BALB/c mice have served as a model to define immunological processes that promote disease [3,6]. An essential parameter that is causatively linked to non-healing *L. major* infections is the production of interleukin (IL)-4. While in self-healing C57BL/6 mice an early *and* transient production of IL-4 in the skin contributes to the IL-12-mediated instruction of Th1 cells [12,13] and to parasite control via dendritic cell and macrophage stimulation [14,15,16], the early *and* sustained release of IL-4 in the draining lymph nodes of *L. major*-infected BALB/c mice [17,18,19] in conjunction with the activation of the NLRP3 (nod-like receptor protein 3) inflammasome [20] is decisive for the differentiation and expansion of Th2 cells. Th2 cells, in turn, cause alternative macrophage activation, which is characterized by the upregulation of arginase 1 and the inhibition of NOS2 enzyme activity due to the depletion of the joint substrate L-arginine, resulting in an impaired killing of *Leishmania* and the progression of disease [21,22]. The key cell types that account for the early IL-4 production in *L. major*-infected BALB/c or C57BL/6 mice have been a matter of controversy [13,17,23,24].

Innate lymphoid cells (ILC) are CD45^+^ cells of hematopoietic origin that have a lymphocyte-like morphology but lack adaptive antigen receptors and classical cell lineage markers [25]. Based on their transcription factor usage and the production of different sets of cytokines, ILC are divided into three main subsets: ILC1 (expression of the transcription factor T-bet, production of IFN-γ and TNF), ILC2 (expression of GATA3; production of IL-4, IL-5, IL-9 and IL-13) and ILC3 (expression of RORγt; production of IL-17A and IL-22). ILC2 reside in mucosal tissues and the dermis but also migrate via the blood and lymph [26,27]. They are phenotypically characterized by a variety of cell surface molecules (e.g., CD90 (Thy1), CD25 (IL-2Rα), CD127 (IL-7Rα), killer cell lectin-like receptor subfamily G member 1 (KLRG1), stem cell antigen (Sca)-1, IL-25 receptor, IL-33 receptor (ST2), programmed death-1 (PD-1)) [28]. Activation of ILC2 by IL-18, IL-25, IL-33 or thymic stromal lymphopoietin (TSLP) induces the release of Th2-like cytokines (e.g., IL-4, IL-5, and IL-13) or IL-10 [29,30,31,32]. In contrast, exposure of ILC2 to type I interferons (IFN-α/β), IFN-γ or IL-27 strongly impeded their production of type 2 cytokines [29,33,34]. Due to their cytokine expression pattern, ILC2 are central regulators of the immune response against helminths, where they mediate the expulsion of worms and tissue repair, but are also involved in pathological processes such as tissue fibrosis and the initiation and exacerbation of allergies [35,36,37,38,39]. Whether ILC2 contribute to the initiation of a protective Th1 or a disease-mediating Th2 response in *L. major*-infected mice, has not yet been investigated.

In the present study, we addressed the question whether ILC2 are an integral part of the innate immune response to *L. major* in the skin of infected mice. To this end, we analyzed the prevalence of ILC2 in the dermis of naïve versus *L. major*-infected BALB/c and C57BL/6 mice. We also studied the expression of ILC2-activating and ILC2-inhibiting cytokines following infection and tested whether ILC2 are early IL-4 producers during *L. major* infection. Our results show that ILC2 do not expand in response to *L. major* infection and are not a predominant source of IL-4 during the first 5 to 6 days of infection.

## 2. Results

### 2.1. Prevalence of ILC2 in the Skin and Draining Lymph Nodes of Naïve Mice

**Figure 1:**
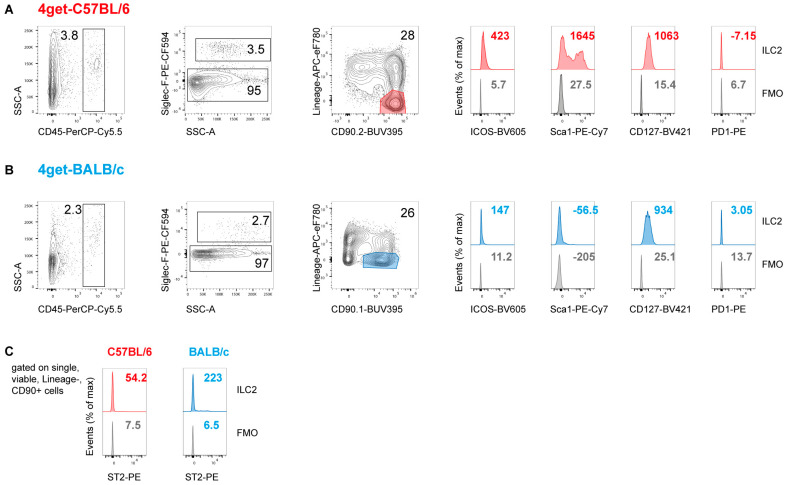
**Detection of ILC2 in the ear skin of naïve C57BL/6 and BALB/c mice.** Flow cytometry of ear skin cells from naïve 4get-C57BL/6 (**A**) and 4get-BALB/c mice (**B**). Lymphomononuclear cells were isolated from ear dermis and epidermis by enzymatic and mechanical dissociation. Single cells were pre-gated on viability and CD45 expression; eosinophils staining positively for SiglecF were excluded. ILC2 were defined as lineage marker-negative, CD90.2^+^ cells. For further characterization, the surface markers CD127, Sca-1, ICOS (CD278), PD1 and ST2 were used. Data are representative of 3 to 4 experiments (**A**,**B**) or 2 experiments (**C**) with 3 to 5 mice pooled per group. The lineage marker-mix included antibodies directed against CD2, CD3, CD5, CD11b, CD11c, CD19, FcεR1a, Ly6G, NKp46 and Ter119. The numbers within the histograms indicate the mean fluorescence intensity of the respective surface markers (**A**–**C**).

In a first set of experiments, we investigated the prevalence of ILC2 in the skin and draining lymph nodes (dLN) of BALB/c and C57BL/6 mice before and after intradermal infection with *L. major* promastigotes. The phenotypical analyses and quantification of dermal ILC2 was performed in IL-4 reporter mice (4get) on a BALB/c or C57BL/6 background [40]. The flow cytometric analysis of dermal cells isolated from naïve ear skin revealed small but distinct populations of cells with an ILC2 phenotype in both strains of mice. ILC2 were defined as CD45^+^CD90.2^+^ cells that were negative for lymphocytic, myeloid and erythroid lineage markers (NKp46, CD2, CD3, CD5, CD19, CD11b, CD11c, FcεR1a, Ly6G and Ter119), following the gating strategy depicted in Figure 1. The ILC2 population accounted for approximately 20–30% of all CD45^+^SiglecF^−^ cells in both C57BL/6 and BALB/c mice. Whereas the ILC2 of both mouse strains were positive for the common ILC marker CD127 (IL-7Rα), C57BL/6 ILC2 showed a partial expression of Sca-1 and of the inducible T-cell costimulator (ICOS or CD278), both of which were only weakly detectable on BALB/c ILC2. Resting ILC2 of naïve C57BL/6 and BALB/c mice were negative for PD-1, confirming that PD-1 is primarily expressed by activated ILC2 [41]. (Figure 1A,B). In accordance with previous data on the phenotype of skin-derived ILC2 [42], the dermal ILC2 of both C57BL/6 and BALB/c mice were also negative for ST2 (Figure 1C). The detectable expression of GATA3 by the lineage-negative CD90.2^+^ ILC2 (Supplementary Figure S1) ranged from ~17% to ~50% (BALB/c: *n* = 5; C57BL/6: *n* = 2), which presumably was due to a variable sensitivity of the intracellular staining.

**Figure 2:**
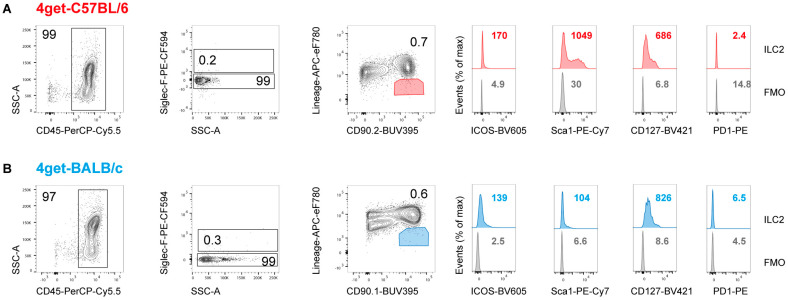
**Lymph nodes draining the ear skin of naïve C57BL/6 and BALB/c mice contain only a small population of ILC2.** Flow cytometry of lymphomononuclear cells isolated from C57BL/6 (**A**) and BALB/c 4get mice (**B**). Single cells were pre-gated on viability and CD45 expression; eosinophils staining positively for SiglecF were excluded. ILC2 were defined as lineage marker-negative, CD90.2^+^ cells. For further characterization, the surface markers CD127, Sca-1, ICOS (CD278) and PD1 were used. Data are representative of 3 to 4 experiments with 3 to 5 mice pooled per group. Lineage-mix included antibodies directed against CD2, CD3, CD5, CD11b, CD11c, CD19, FcεR1a, Ly6G, Nkp46 and Ter119. The numbers within the histograms indicate the mean fluorescence intensity of the respective surface markers (**A**,**B**).

The flow-cytometric analysis of LNs draining the ear skin of naïve C57BL/6 and BALB/c mice yielded only few cells (<1%) with an ILC2 phenotype (lineage marker-negative, CD90^+^CD127^+^SiglecF^−^CD45^+^). The expression of Sca-1, ICOS and PD1 by dLN ILC2 of C57BL/6 and BALB/c mice resembled the pattern seen in dermal ILC2 (Figure 2). As ILC2 were readily detectable in the ear skin, whereas their prevalence was low in the dLNs, we restricted our further analyses to the skin, also because this is the primary site of contact between *Leishmania* parasites and immune cells.

### 2.2. Prevalence of ILC2 in the Skin of L. major-Infected Mice

**Figure 3:**
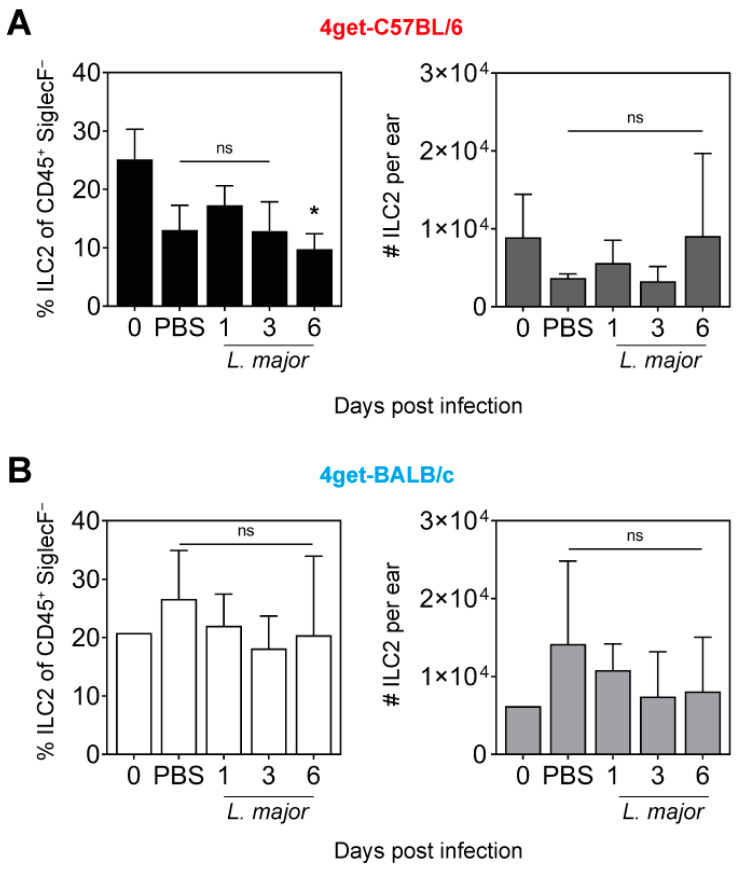
**Infection with *L. major* induces only minor changes in the frequency and absolute numbers of dermal ILC2.** Flow cytometry (see Figure 1) of ear skin cells from naïve (d0), PBS-injected (d1) or *L. major*-infected (1 × 10^5^ promastigotes intradermally; d1, 3 and 6 p.i.) of 4get-C57BL/6 (**A**) and 4get-BALB/c mice (**B**). Data show mean ± SD of 3 to 4 independent experiments. For each time point, cells from 3 to 4 mice were pooled. Statistical significance was determined using Kruskal–Wallis test followed by Dunn‘s multiple comparisons correction against the control (d0). ns *p* > 0.05; * *p* < 0.05.

In order to investigate possible changes in the frequencies and absolute numbers of ILC2 during the course of *L. major* infection, C57BL/6 and BALB/c mice were infected intradermally into the ear skin with 1 × 10^5^ *L. major* promastigotes and were analyzed at different time points post infection (p.i.). using flow cytometry. Whereas in naïve C57BL/6 mice, the CD45^+^ cell population contained around 25% lineage marker-negative CD90^+^ ILC2, the frequencies (in %) of ILC2 were tentatively lower in both PBS-treated as well as in infected mice at d1 and d3 p.i., reaching a significantly reduced level at d6 p.i. (Figure 3A, left panel). The absolute numbers of ILC2 did not significantly vary during infection with *L. major* and showed a maximum of approximately 9 × 10^3^ cells per ear at d6 p.i. (Figure 3A, right panel). In uninfected BALB/c mice, approximately 20% of all CD45^+^ cells were ILC2. Infection with *L. major* did not cause significant changes in the percentages of ILC2 compared to naïve mice (Figure 3B, left panel). Consequently, the numbers of ILC2 in naïve BALB/c mice or in BALB/c mice treated with PBS or infected with *L. major* remained in the same order of magnitude (Figure 3B, right panel). In one experiment using both mouse strains, staining of lineage marker-negative CD90^+^ ILC2 for the cell division marker Ki67 did not provide evidence that *L. major* infection caused ILC2 proliferation (C. Sasse, data not shown). Thus, the number of dermal ILC2 was relatively low (around 1 × 10^4^ per ear) in both mouse strains and did not expand in response to *L. major* parasites during the first six days of infection.

### 2.3. Expression of Cytokines Regulating ILC2 Activity in the Ear Skin of L. major-Infected Mice

**Figure 4:**
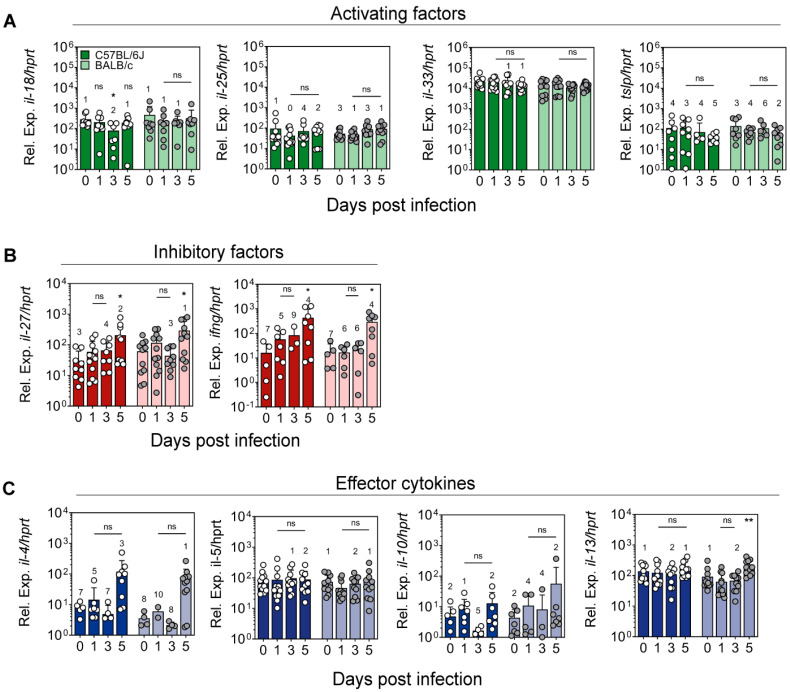
**mRNA expression of cytokines that modulate ILC2 or are produced by ILC2 in the ear skin of naïve or *L. major*-infected mice**. C57BL/6J (dark colors in all panels) and BALB/c mice (light colors in all panels) were infected intradermally with 1 × 10^5^ *L. major* promastigotes into the ear skin. RT-PCR was used to quantitate the cytokine mRNA levels that were normalized to the expression of the house-keeping gene hprt. Data show mean ± SD of 4 experiments with 3 mice per time point and group, resulting in up to 12 samples in total per time point. Numbers above the bars indicate the number of samples where gene expression was undetectable. Dependent on the distribution of data, statistical significance was determined by one-way ANOVA followed by Holm–Sidak’s multiple comparisons test or Kruskal–Wallis test followed by Dunn’s multiple comparisons test, comparing each time point with the control (d0). ns *p* > 0.05; * *p* < 0.05; ** *p* < 0.01 (**A**–**C**).

Having seen that the numbers of ILC2 in the ear skin remained more or less constant after *L. major* infection, we next addressed the question whether the immune response to the parasite might alter the activation status of ILC2. To this end, we analyzed the mRNA expression of cytokines that (a) are known to stimulate (e.g., IL-18, IL-25, IL-33 and TSLP) or inhibit (e.g., type I IFNs, IFN-γ and IL-27) the proliferation and activity of ILC2 or (b) are typical secretory products of activated ILC2 (e.g., IL-4, IL-5, IL-10 and IL-13). Quantitative RT-PCR analysis of RNA samples from naïve or *L. major*-infected whole ear tissue revealed that the mRNA levels of IL-18, IL-25 and TSLP were not significantly higher in infected tissue (d1, 3 and 5 p.i.) compared to naïve tissue in both C57BL/6 and BALB/c mice. IL-33, which was already prominently expressed in naïve ear skin, was also not upregulated upon infection with *L. major* in either mouse strain (Figure 4A). With respect to inhibitory cytokines, the expression of IL-27 mRNA was significantly higher at d5 p.i. compared to uninfected skin in both C57BL/6 and BALB/c mice. The gene expression of IFN-γ followed a similar course, with statistically significantly higher levels seen at d5 p.i. in both C57BL/6 and BALB/c mice (Figure 4B). Concerning the effector products of ILC2, a 15- to 20-fold increase in IL-4 gene expression was seen at d5 of *L. major* infection in both C57BL/6 and BALB/c mice, although statistical significance was not yet reached. As previously reported [22], the expression of IL-4 mRNA was comparable in C57BL/6 and BALB/c mice during this early infection period. Unlike IL-4, the levels of IL-5, IL-10 and IL-13 mRNA did not show relevant changes upon infection of the two mouse strains at all time-points analyzed (Figure 4C). From these data, we conclude that an intradermal infection with *L. major* did not elicit major alterations in the expression of cytokines that stimulate ILC2 or support their proliferation. Accordingly, the numbers of ILC2 did not change, and there was only a selective upregulation of type 2 cytokines (IL-4) observed, possibly also resulting from increased levels of IL-27 and IFN-γ.

### 2.4. Cellular Source of IL-4 in the Ear Skin of L. major-Infected Mice

The observation that an intradermal infection with *L. major* led to an upregulation of IL-4 mRNA at d5 of infection raised the question of the cellular origin of IL-4. Considering the notorious difficulty to detect IL-4 in freshly isolated cells ex vivo by intracellular cytokine staining and flow cytometry, we used IL-4 reporter (4get) C57BL/6 and BALB/c mice, in which the IL-4 gene is linked via an internal ribosomal entry site to the gene for enhanced green fluorescent protein (GFP) so that cells with active IL-4 gene transcription show a green fluorescence [40]. As eosinophils have been identified as a source of early IL-4 production in a model of non-healing cutaneous leishmaniasis [24], we included SiglecF as an eosinophil marker in our flow-cytometric analyses.

**Figure 5:**
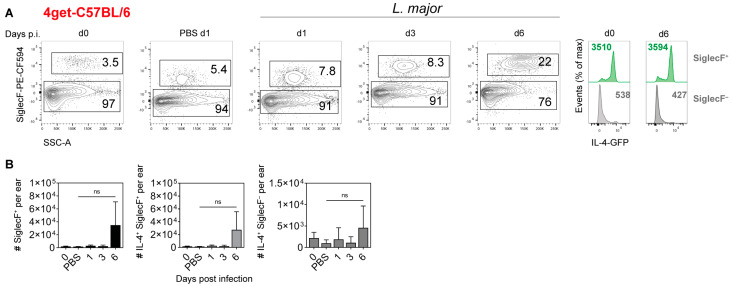
**Detection of IL-4-producing cells in the ear skin of *L. major*-infected C57BL/6 4get mice.** Naïve, PBS-treated or infected C57BL/6 4get mice (1 × 10^5^ *L. major* promastigotes intradermally) were analyzed using flow cytometry at different time points post infection. Single cells were pre-gated on viability and CD45 expression. Eosinophils were defined as CD45^+^SiglecF^+^ cells of high granularity (SSC-A^high^). Frequencies of parent populations are given as numbers inside the gates. Histograms show the expression of IL-4 at d0 and d6 p.i. by SiglecF^+^ and SiglecF^−^ cells. Numbers within the histograms indicate mean fluorescence intensity of the respective surface markers. Graphs are representative of 4 independent experiments (**A**). Numbers of SiglecF^+^ as well as IL-4^+^SiglecF^+^ and IL-4^+^SiglecF^−^ cells per ear were determined. Data show mean ± SD of 4 independent experiments. For each time point, cells from 3 to 4 mice were pooled. Statistical significance was determined using Kruskal–Wallis test followed by Dunn‘s multiple comparisons correction against the control (d0). ns *p* > 0.05 (**B**).

In naïve C57BL/6 ear skin, a small population of SiglecF^+^ cells was detected. In control mice, which were intradermally injected with PBS alone and analyzed one day later, the number of SiglecF^+^ cells was slightly increased. In *L. major*-infected skin, the frequency of SiglecF^+^ cells increased with time, reaching around 22% of all CD45^+^ cells at d6 p.i. At d6 p.i., most SiglecF^+^ cells also expressed GFP, and only a small GFP signal was detectable in the SiglecF^−^ population (Figure 5A). Quantitative analysis revealed that the number of SiglecF^+^ cells per ear increased from d1 to d6 p.i. (Figure 5B, left panel). A similar trend was seen with the number of GFP^+^SiglecF^+^ cells of high granularity (SSC-A^high^), i.e., with the IL-4-producing eosinophils (Figure 5B, middle panel). In contrast, the size of the GFP^+^SiglecF^−^ cell population remained small throughout the entire observation period (Figure 5B, right panel) and contained mast cells (ckit^+^FcεR1a^+^; ≤50%) and basophils (CD200R3^+^CD49b^+^; ≤50%) and, if at all, small quantities of ILC2 (C. Sasse, data not shown).

**Figure 6:**
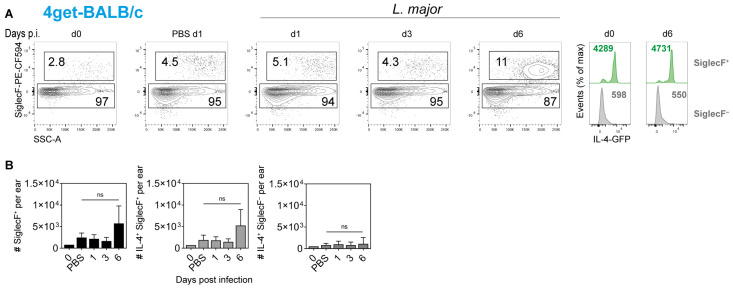
**Detection of IL-4-producing cells in the ear skin of *L. major*-infected BALB/c 4get mice.** Naïve, PBS-treated or infected BALB/c 4get mice (1 × 10^5^ *L. major* promastigotes intradermally) were analyzed using flow cytometry at different time points post infection. Single cells were pre-gated on viability and CD45 expression. Eosinophils were defined as CD45^+^SiglecF^+^ cells of high granularity (SSC-A^high^). Frequencies of parent populations are given as numbers inside the gates. Histograms show the expression of IL-4 at d0 and d6 p.i. by SiglecF^+^ and SiglecF^−^ cells. Numbers within the histograms indicate the mean fluorescence intensity of the respective surface markers. Graphs are representative of 3 independent experiments (**A**). Numbers of SiglecF^+^ as well as IL-4^+^SiglecF^+^ and IL-4^+^SiglecF^−^ cells per ear were determined. Data show mean ± SD of 3 independent experiments. For each time point, cells from 3 to 4 mice were pooled. Statistical significance was determined using Kruskal–Wallis test followed by Dunn‘s multiple comparisons correction against the control (d0). ns *p* > 0.05 (**B**).

Equivalent analyses were performed with BALB/c 4get mice. Again, the analysis of naïve and *L. major*-infected ear skin revealed a slight increase in SiglecF^+^ cells upon injection of PBS or infection with *L. major* at d1 after infection. By d6 p.i., the frequency of SiglecF^+^ cells had doubled and reached 11% of all CD45^+^ cells. All SiglecF^+^ cells also expressed GFP at d6 p.i., whereas only low GFP expression levels was detectable in the SiglecF^−^ compartment (Figure 6A). Quantification of SiglecF^+^ and IL-4^+^SiglecF^+^ cells showed an increase in these cells per ear at d6 p.i., which, however, did not reach statistical significance (Figure 6B, left and middle panel). The number of IL-4^+^SiglecF^−^ cells per ear was small, did not increase upon infection with *L. major* (Figure 6B, right panel) and consisted mostly of mast cells and basophils (*C.* Sasse, data not shown).

Together, the use of IL-4 reporter mice (BALB/c and C57BL/6 4get mice) revealed an increase in eosinophils (i.e., SiglecF^+^CD45^+^SSC-A^high^ cells), which reached up to 3.5 × 10^4^ cells per ear in C57BL/6 and 6 × 10^3^ cells per ear in BALB/c mice at d6 after infection. In both mouse strains, the number of GFP^+^SiglecF^+^ IL-4-producing cells mirrored this increase in absolute SiglecF^+^ cell numbers. As only a small fraction of CD45^+^SiglecF^−^ cells expressed GFP in both C57BL/6 and BALB/c 4get mice, we conclude that eosinophils rather than ILC2 account for the observed increase in IL-4 expression early after infection with *L. major*.

Finally, in independent experiments, we evaluated whether the results obtained in the intradermal ear infection model also held true when a high dose of *L. major* promastigotes (3 × 10^6^) was injected subcutaneously into the foot skin of C57BL/6 4get mice. Again, at different time points of infection (e.g., 12 h, d5 and d6) we observed that the expression of IL-4 was primarily found in CD45^+^SiglecF^+^ eosinophils and some other myeloid cells, but not in CD45^+^SiglecF^−^ ILC2 (D. Barinberg, S. Obermeyer and U. Schleicher, unpublished observation).

## 3. Discussion

In the past, only few studies have investigated the prevalence or possible function of ILC in the context of *Leishmania* infections. Rodriguez et al. studied the distribution of ILC1, ILC2 and ILC3 in the peripheral blood of Venezuelan patients with localized (LCL; *n* = 7), intermediate type (ICL; *n* = 3) or diffuse cutaneous leishmaniasis (DCL; *n* = 3) or with mucocutaneous leishmaniasis (MCL; *n* = 10) as compared to healthy controls (*n* = 17). Although the underlying parasite species was not reported and the number of analyzed patients was small, the authors obtained evidence that ILC1 were more frequent in LCL patients, whereas ILC2 were the predominant ILC type in DCL patients, who exhibit T cell anergy and disseminated disease [43]. Singh et al. reported that IL-17A-producing ILC3 were enriched in the skin lesions of C57BL/6 mice at day 7 of infection with *L. major*. Colonization of the mouse skin with the bacterial species *Staphylococcus (S.) epidermidis* (human commensal) or *S. xylosus* (mouse commensal) prior to *L. major* infection caused an increase in lesion size, which was paralleled by a ≤ two-fold increase in the number of infiltrating ILC3, suggesting that microbiota-driven ILC3 contributed to disease development [44]. In the present study, we first showed that ILC2 were readily detectable in the dermis of naïve and *L. major*-infected mice but did not expand following infection, neither in self-healing C57BL/6 nor in non-healing BALB/c mice. Second, we found that *L. major* infection did not alter the mRNA expression of various ILC2-activating or ILC2-inhibiting cytokines. The quantitative gene expression analysis was performed with whole ear tissue samples rather than sorted ILC2 so that subtle changes in the cytokine expression might have escaped detection. Finally, we demonstrated that eosinophils rather than ILC2 were the predominant source of IL-4 during the innate phase of *L. major* infection.

In the past, several types of cells were identified or proposed to serve as early IL-4 producers during *L. major* infection of either BALB/c or C57BL/6 mice. These included NK1.1^−^Vα8β4^+^CD4^+^ T cells in the draining lymph node [45] as well as keratinocytes [13], neutrophils [23] and eosinophils at the dermal site of infection [24]. The functional consequences of early IL-4 production during *L. major* infection are diverse, depending on the time point of infection, the analyzed tissue, the studied mouse strain and the used parasite strain. In the case of BALB/c mice and the *L. major* LV39 or FEBNI strains, IL-4 production by neutrophils or NK1.1^−^Vα8β4^+^CD4^+^ T cells in the skin-draining lymph nodes was suggested to promote the development of disease-mediating Th2 cells, e.g., by downregulating the expression of the IL-12Rβ2 chain [17,18,23]. However, in the same experimental mouse system, Hurdayal et al. observed that endogenous IL-4 also conveys disease-ameliorating effects via promoting IL-12 production by CD11c^+^ dendritic cells [15]. Likewise, in C57BL/6 mice infected with the *L. major* LV39 or the *L. major* FEBNI strain, the early and transient peak of IL-4, presumably generated by epidermal keratinocytes, contributed to the instruction of Th1 cells via upregulation of IL-12 [13]. Finally, C57BL/6 mice infected with the *L. major* Seidman strain showed a progressive course of infection, despite the development of Th1 cells [46]. In this particular model of non-healing cutaneous leishmaniasis, IL-4 released by eosinophils induced a phenotype of alternative (M2-like) activation in dermal macrophages and thereby generated a replication niche for the parasites. The macrophages, in turn, released the chemokine CCL24, which further stimulated the influx of eosinophils and their interaction with macrophages in the skin [24]. The clinical relevance of the early interaction between IL-4-producing eosinophils and dermal macrophages became apparent by the ameliorated disease that occurred in *L. major* Seidman-infected bone marrow chimeric mice lacking IL-4 expression in the innate immune compartment or in mice with an eosinophil-specific deletion of IL-4 and IL-13 [24]. Our present finding that eosinophils are a source of IL-4 in C57BL/6 and BALB/c mice infected with the *L. major* FEBNI strain is in line with the observation by Lee et al. [24].

Prior to the research by Lee et al. [24] and our present work, other groups had already reported on the accumulation of eosinophils at the skin site early or late after infection of inbred (BALB/c or C57BL/6) or outbred mice with *L. major* [47] or other *Leishmania* species causing cutaneous disease, notably *L. mexicana* and *L. amazonensis* [48,49,50,51,52]. These studies focused on the immunopathological analysis of the skin lesions and provided limited evidence for a potential antileishmanial effect of eosinophils. The latter was suggested by (a) the rare observation of intact or partly degraded *L. major* amastigotes within eosinophils [47,49]; (b) the close neighborhood of eosinophils (that contain granules with toxic effector molecules) to free parasites or infected macrophages in acute or chronic lesions [47,51,52]; and (c) by the principal ability of rat peritoneal eosinophils to kill extracellular or intracellular *L. major* of *L. amazonensis* parasites in vitro in an iNOS/NO- or granule-dependent manner [53,54]. Clearly, the elegant work of Lee et al. on the deactivation of macrophages by eosinophil-derived IL-4 following *L. major* Seidman infections of C57BL/6 mice does not discount the possibility of a protective effect of eosinophils in mouse models with other *L. major* strains or different *Leishmania* species. In this context, IL-4 (derived from eosinophils or other cell types) can support the antileishmanial activity of macrophages [14,55] as well as of eosinophils [56].

In conclusion, the presented data show that eosinophils, but not ILC2, form a major source of IL-4 during the early phase of *L. major* infection of mice. Thus, it is unlikely that ILC2 are involved in the early IL-4-dependent instruction of Th1 cells or the development of Th2 cells after *L. major* infection. Future studies with ILC2-deficient mice need to address the question whether dermal ILC2 nevertheless exert functions during the early or late phase of *L. major* infections. For example, our current data do not exclude the possibility that ILC2—by virtue of their constitutive expression of IL-5 [57]—contribute to the influx of eosinophils seen after *L. major* infection. As ILC2 are not only producers of Th2-like cytokines that support the development of a non-protective immune response to *L. major*, but are also involved in tissue repair, we are particularly interested in a potential role of ILC2 during the resolution phase of cutaneous leishmaniasis.

## 4. Materials and Methods

### 4.1. Mice

Female C57BL/6J and BALB/c (Charles River, Sulzfeld, Germany) as well as IL-4/eGFP 4get reporter mice on a C57BL/6 and BALB/c background [40] (kindly provided by D. Voehringer, Erlangen, Germany) were used between 6–14 weeks of age for infection experiments. Mice were kept under specific pathogen-free conditions. Animal care and experiments were performed in accordance with German regulations after being approved by the local governments (Regierung von Mittelfranken, Ansbach, Germany Az.: DMS-2532-2-25; Regierung von Unterfranken, Würzburg, Germany, Az.: RUF-55.22-2532-2-1461-14).

### 4.2. Parasites and Infection

For infection, stationary-phase *L. major* promastigotes (strain MHOM/IL/81/FEBNI) were used. Origin and culturing of the parasites were described elsewhere [6,58]. Mice were either infected intradermally (i.d.) into the skin of both ears with 1 × 10^5^ parasites in 20 µL PBS or subcutaneously (s.c.) into the skin of both hind feet with 3 × 10^6^ parasites in 50 µL PBS. Control mice were either left untreated (naïve) or injected with the respective amount of PBS.

### 4.3. Preparation of Single Cell Suspensions

*Ear skin*: Dermal and epidermal layer of each ear were separated, cut into small pieces and digested in RPMI 1640 medium (Gibco™ Life Technologies; ThermoFisher Scientific, Waltham, MA, USA, cat. no. 21875-034), which was supplemented with 1 mg/mL DNase I and 0.25/mL mg Liberase^TM^ (both Sigma-Aldrich, St. Louis, MO, USA), at 37 °C under gentle vibration for 1 h. Digested tissue was sequentially passed through a 100, 70 and 40 µm cell strainer in order to remove cell debris.

*Foot skin*: Whole foot skin was cut into small pieces, which were digested for 45–60 min at 37 °C under gentle vibration in RPMI 1640 containing 0.2 mg/mL collagenase P and 0.1 mg/mL DNAse I (both Roche, Basel, Switzerland). Cell suspensions were passed through a 70 µm cell strainer, resuspended in 2.5 mL of 40% Percoll^®^ preparation (20 mL Percoll^®^; GE Healthcare, Chicago, IL, USA), 2.2 mL 10× PBS, 27.8 mL HBSS without Ca^2+^ and Mg^2+^ (Sigma-Aldrich, St. Louis, MO, USA) and layered onto 1.5 mL 60% Percoll^®^ (30 mL Percoll^®^, 3.3 mL 10× PBS, 16.7 mL HBSS). After centrifugation for 20 min at 931× *g* and room temperature, the cell layer at the interface was collected, washed and resuspended in PBS.

*Draining lymph node (dLN)*: The dLN was cut into small pieces and digested as described for the foot skin. Digested tissue was passed through a 70 µm cell strainer.

### 4.4. Flow Cytometry

Cells resuspended in PBS were stained with the fixable cell viability dye eFluor 506 (eBioscience, Thermo Scientific, Waltham, MA, USA) according to the manufacturer’s protocol and washed with PBS/1% FCS/5 mM EDTA. After incubation with TruStain fcX α-mouse CD16/32 blocking antibody (BioLegend, San Diego, CA, USA), staining of different surface markers (see Table 1) was performed for 20 min at 4 °C followed by a washing step with PBS/1% FCS.

For intracellular staining cells were fixed with Foxp3/Transcription Factor Staining Buffer Set (eBioscience, Thermo Fisher Scientific, Waltham, MA, USA) for 30 min at 4 °C, washed with 1× Perm Buffer and stained for transcription factors (GATA3) (see Table 1) for 30 min at 4 °C in 1× Perm Buffer.

### 4.5. Quantitative Real-Time PCR

Steel beads were added to frozen tissue, and tissue was homogenized with the help of a tissue homogenizer. RNA was extracted using the peqGold TriFast^TM^ reagent (Peqlab, VWR, Radnor, PA, USA). Then, 3 to 5 µg RNA was reverse transcribed using the High Capacity cDNA Reverse Transcription Kit (ThermoFisher Scientific). qPCR with gene-specific assays (TaqMan Gene Expression Assays, Thermo Fisher Scientific; see Table 2 below) was performed on the Viia7 Real-Time PCR System (ThermoFisher Scientific, Waltham, MA, USA) using 20 to 100 ng of cDNA. Mouse hypoxanthine guanine phosphoribosyl transferase-1 (Hprt-1) was used as housekeeping gene for normalization. mRNA levels were calculated by the following formula: relative expression = 2^−(CT(Target)−CT(Endogenous control))^ × f, with f = 10^4^ as an arbitrary factor.

## Figures and Tables

**Table 1:** Antibodies and reagents used for flow cytometric analysis.

Antibody	Source	Order No.	Batch, Clone	Fluorochrome Conjugation	Dilution
Rat anti-mouse CD2	BioLegend	100104	RM2-5	Biotin	1:100
Hamster anti-mouse CD3	BioLegend	100304	145-2C11	Biotin	1:100
Rat anti-mouse CD5	BioLegend	100604	53–7.3	Biotin	1:100
Rat anti-mouse CD11b	BioLegend	101204	M1/70	Biotin	1:100
Hamster anti-mouse CD11c	BD	553800	HL3	Biotin	1:100
Rat anti-mouse CD19	BioLegend	115504	6D5	Biotin	1:100
Rat anti-mouse CD45	BioLegend	103132	30-F11	PerCP-Cy5.5	1:100
Hamster ant-mouse CD49b	BD	740250	HMα2	BUV395	1:100
Mouse anti-mouse CD90.1	BD	740261	OX-7	BUV395	1:300
Rat anti-mouse CD90.2	BD	565257	53–2.1	BUV395	1:300
Rat anti-mouse CD117 (ckit)	BioLegend	105812	2B8	APC	1:100
Rat anti-mouse CD127	BioLegend	135027	A7R34	BV421	1:100
Rat anti-mouse CD170 (SiglecF)	BioLegend	61-1702-82	1RNM44N	PE-Dazzle594	1:200
Rat anti-mouse CD200R3	BioLegend	142206	Ba13	PE	1:100
Hamster anti-mouse CD278 (ICOS)	BioLegend	313538	C398.4A	BV605	1:100
Rat anti-mouse CD279 (PD-1)	BioLegend	135205	29F.1A12	PE	1:100
Rat anti-mouse CD335 (Nkp46)	BioLegend	137616	29A1.4	Biotin	1:100
Rat anti-mouse F4/80	BioLegend	123114	BM8	PE-Cy7	1:100
Hamster anti-mouse FcεR1α	BioLegend	134304	MAR-1	Biotin	1:100
Rat anti-mouse/human GATA3	ebiosciences	12-9966-42	TWAJ	PE	5 µL/test
Rat anti-mouse Ly6A/E (Sca-1)	BD	561021	D7	PE-Cy7	1:100
Rat anti-mouse Ly6G	BioLegend	127604	1A8	Biotin	1:100
Rat anti-mouse ST2 (IL-33R)	MD Bioproducts	101001PE	DJ8	PE	1:100
Rat anti-mouse Ter-119	BD	553672	Ly-76	Biotin	1:100
Streptavidin	ebiosciences	47-4317	N/A	APC-eFluor™ 780	1:100

**Table 2:** Gene-specific assays used for qRT-PCR.

Gene	Assay ID
Hprt	Mm00446968_m1
Ifng	Mm00801778_m1
Il4	Mm00445259_m1
Il5	Mm00439646_m1
Il10	Mm01288386_m1
Il13	Mm00434204_m1
Il18	Mm00434225_m1
Il25	Mm00499822_m1
Il27	Mm00461164_m1
Il33	Mm00505403_m1
TSLP	Mm01157588_m1

## Data Availability

All data mentioned are presented within this article.

## Supplementary Materials

The following supporting information can be downloaded at: https://www.mdpi.com/article/10.3390/pathogens11080828/s1, Figure S1: Detection of GATA3 in the CD45^+^CD90^+^Lin^−^ ILC2 population in the ear skin of naïve C57BL/6 and BALB/c mice.
