# Supplementary material for: Eosinophils, but Not Type 2 Innate Lymphoid Cells, Are the Predominant Source of Interleukin 4 during the Innate Phase of Leishmania major Infection
Source: Pathogens. 2022 Jul 25;11(8):828. doi: 10.3390/pathogens11080828 (PMC9331382; doi:10.3390/pathogens11080828)
Supplement: Supplementary file 1 [file pathogens-11-00828-s001.zip › pathogens-1796795-supplementary.pdf]

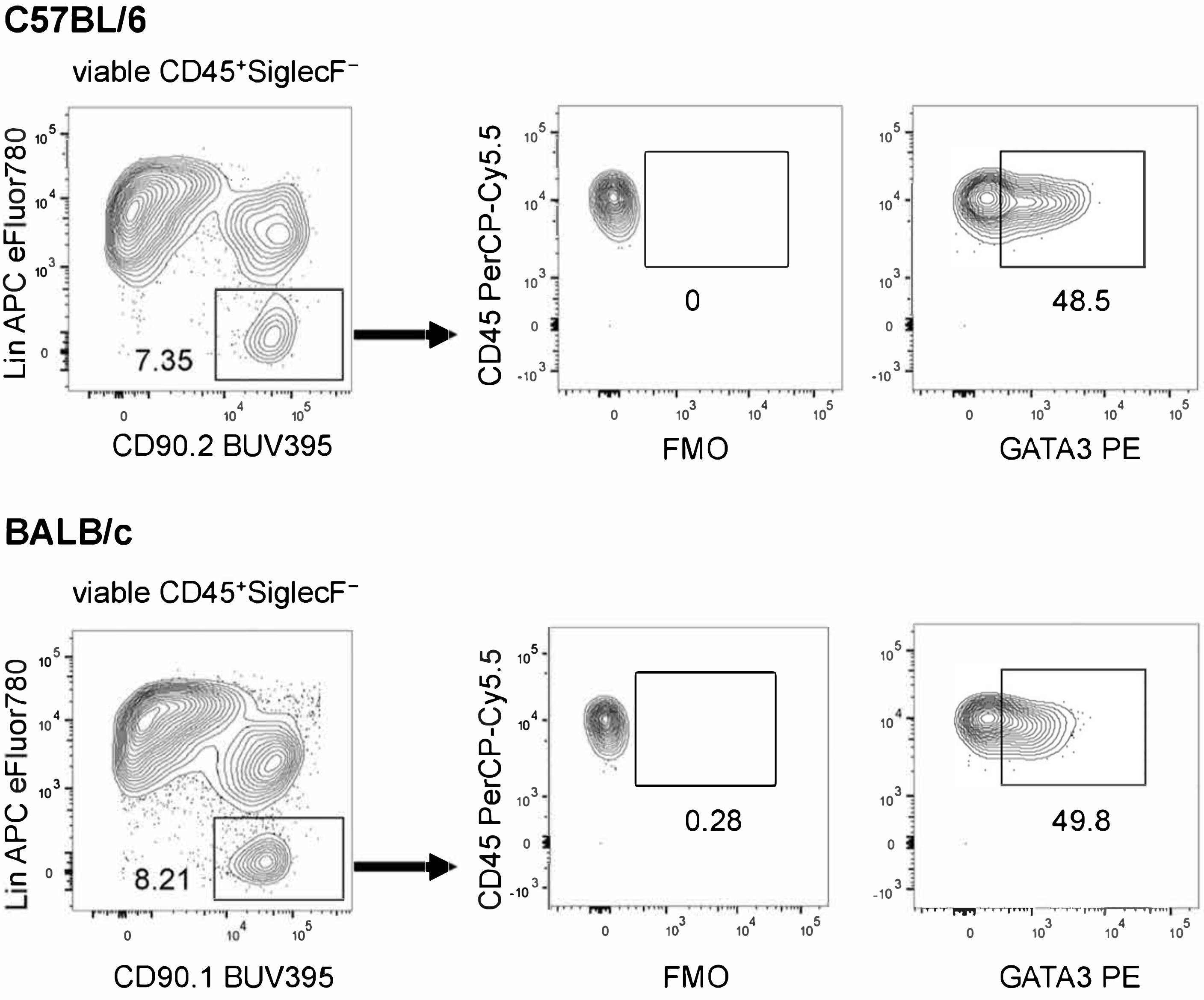

Supplemental Figure S1. Detection of GATA3 in CD45$^+$CD90$^+$Lin$^-$ ILC2 population in the ear skin of naïve C57BL/6 and BALB/c mice. Flow cytometry of ear skin cells from naïve C57BL/6 and BALB/c mice. Lymphomononuclear cells were isolated from ear dermis and epidermis by enzymatic and mechanical dissociation. Single cells were pre-gated on viability and CD45 expression; eosinophils staining positively for SiglecF were excluded. ILC2 were defined as lineage marker-negative, CD90$^+$ cells. To further verify the ILC2 phenotype, intracellular staining of the transcription factor GATA3 was performed. Data is representative of 2 (C57BL/6) or 5 (BALB/c) experiments with 3 to 5 mice pooled per group. The lineage marker-mix included antibodies directed against CD2, CD3, CD5, CD11b, CD11c, CD19, FceR1a, Ly6G, NKp46 and Ter119. The numbers indicate the percentage of the gated population.
